# Turkish validity and reliability of Eustachian tube dysfunction questionnaire-7^☆^

**DOI:** 10.1016/j.bjorl.2017.05.001

**Published:** 2017-05-31

**Authors:** Erdoğan Özgür, Cem Bilgen, Beyhan Cengiz Özyurt

**Affiliations:** a Nazilli State Hospital, Otorhinolaryngology Clinic, Aydın, Turkey; b Ege University, Department of Otorhinolaryngology, Izmir, Turkey; c Celal Bayar University, Department of Public Health, Manisa, Turkey

**Keywords:** Eustachian tube, Reliability and validity, Validation studies, Tuba auditiva, Confiabilidade e validade, Estudos de validação

## Abstract

**Introduction:**

During clinical evaluations, in order to interpret patients’ complaints caused by Eustachian tube dysfunction and to monitor the success of the treatment, standardized and disease-related scales are necessary.

**Objective:**

The aim of this study was to investigate the validity and reliability of the Turkish version of Eustachian tube dysfunction questionnaire-7.

**Methods:**

Forty patients diagnosed with Eustachian tube dysfunction and 40 healthy individuals were enrolled for the study. After language validation of the Eustachian tube dysfunction questionnaire-7 for Turkish, a scale was completed by the both Eustachian tube dysfunction and control groups. Two weeks after the first evaluation, 15 of the cases filled out the scale again without any treatment intervention. Known-groups method was used in validity analysis. Floor-ceiling effect, test–retest method, item-total score correlation and internal consistency analysis were used in reliability analyses.

**Results:**

Cronbach's alpha coefficient was 0.714 for the entire questionnaire. The test–retest reliability coefficient for the total scale was determined as 0.792, indicating correlation between the two questionnaires completed by the same patient over time. In the Eustachian tube dysfunction group, total and each item scores were found significantly higher than the control group (*p* < 0.001).

**Conclusion:**

The Turkish version of Eustachian tube dysfunction questionnaire-7 was found to be highly valid and reliable. This scale is recommended to use for screening of Eustachian tube dysfunction and evaluating treatment outcome.

## Resumo

**Introdução:**

No intuito de interpretar as queixas dos pacientes causadas por disfunções na tuba auditiva durante uma avaliação clínica, e para monitorar o sucesso do tratamento, há necessidade de escalas padronizadas relacionadas à doença.

**Objetivo:**

Investigar a validade e a confiabilidade da versão turca do Eustachian tube dysfunction questionnaire-7.

**Método:**

Quarenta pacientes diagnosticados com disfunção da tuba auditiva e 40 indivíduos saudáveis foram incluídos no estudo. Após a validação do Eustachian tube dysfunction questionnaire-7 para o idioma turco, o questionário foi aplicada aos grupos disfunção da tuba auditiva e controle. Duas semanas após a primeira avaliação, 15 pacientes preencheram o questionário novamente sem qualquer tratamento. O método de grupos conhecidos foi utilizado na análise de validade. Os efeitos teto e chão, o método teste-reteste, a correlação se escore de item-total e a análise de consistência interna foram utilizados nas análises de confiabilidade.

**Resultados:**

O coeficiente alfa de Cronbach foi de 0,714 para todo o questionário. O coeficiente de confiabilidade teste-reteste para a escala total foi determinado como 0,792, indicando correlação entre os dois questionários preenchidos pelo mesmo paciente ao longo do tempo. No grupo disfunções da tuba auditiva, foi observado que os escores total e de cada item foram significativamente maiores do que no grupo controle (*p* < 0,001).

**Conclusão:**

A versão no idioma turco do Eustachian tube dysfunction questionnaire-7 foi considerada altamente válida e confiável. Recomenda-se a utilização dessa escala para a triagem de disfunções da tuba auditiva e avaliação do resultado do tratamento.

## Introduction

Beyond just being a tube that links two anatomic spaces, the Eustachian tube possesses crucial functions for the middle ear cavity as such as ventilating, regulating its pressure and protection.^1^ Once these functions are disrupted Eustachian tube dysfunction (ETD) occurs. This condition is common in otorhinolaryngology practice.^2^ In the beginning, ETD causes complaints such as mild aural fullness. However, as the situation gets more obstinate it may induce tympanic membrane retractions, adhesions, recurrent otitis media with effusion and even chronic otitis media. Hence, diagnosis and treatment for chronic ETD is critically important.^3^ During clinical applications, in order to interpret patients’ complaints caused by ETD and to monitor the success of the treatment, it is required to have standardized and disease-related scales. For this purpose, McCoul et al. published ‘Eustachian tube dysfunction questionnaire-7 (ETDQ-7)’ as a valid and reliable method for clinical applications.^4^ This scale has been used to assess the effectiveness of the treatment as well as determining the severity of disease.^5^, ^6^, ^7^ The aim of this study was to investigate the validity and reliability of the Turkish version of ETDQ-7.

## Methods

This study was designed as a validation study. This study was in accordance with Declaration of Helsinki and has been approved by the Local Ethics Committee (20478486-348). All participants were fully informed about the study and written informed consent forms were obtained. To carry out the validity and reliability of Turkish version of ETDQ-7, permission was obtained from the author of the original scale (Anand, V.).

### Selection of participants

In validity and reliability studies, it is suggested that sample size is required to be at least 5–10 fold more than number of items.^8^, ^9^

### Selection of the case group

A total of 40 individuals (older than 18 years old) admitted to outpatient clinic of otorhinolaryngology between August 2015 and December 2015 and were diagnosed with ETD were enrolled for the ETD group. Participants who had chronic diseases like diabetes mellitus, hypertension, atherosclerosis, or were diagnosed with active upper respiratory infection, tympanic membrane perforation, hearing loss and/or tinnitus in Ear, Nose and Throat (ENT) examination and failed to fill ETDQ-7 form were excluded from the study.

### Selection of the control group

Forty healthy, age and sex-matched adults were included as a control group. It was proven with medical examination and audiological methods that the control group did not have ETD. Inclusion criteria for the control group were listed as answering all scale questions, not having any acute or chronic sinonasal pathology, any acute or chronic otitis media, tinnitus, hearing loss or obstructive sleep apnea during ENT examination. Afterwards, patient data sheet related to demographic features and ETDQ-7 forms were applied to all control group.

### Detecting Eustachian tube dysfunction

Routine ENT examination was performed to the individuals. After that, tympanometric analysis and Williams test were conducted (Interacoustics AZ 26 clinical impedance audiometer, Assens, Denmark). Results obtained from Williams test were recorded for each ear as P1, P2 and P3. If pressure differences between P1–P2 and P2–P3 were less than 10 daPa or pressure difference between Pmax − Pmin was less than 15 daPa, the test subject was considered to have ETD.^10^, ^11^

### Eustachian tube dysfunction questionnaire-7

Based on symptoms of ETD, this Likert-type scale consisted of seven questions, with a response of “1” indicating no problem and “7” indicating a severe problem. When an overall score got higher, it was considered to be an increase in the severity of the disease. In ETDQ-7 patients were asked if they had pressure, pain in the ears, a feeling of clogged or muffled hearing, ear symptoms during sinusitis or common cold, crackling sounds or tinnitus in one or both ears over the previous one month period. The lowest total score was 7 while highest was 49 in this scale.^4^

### Procedure

Internationally accepted steps defined in related methods were used during language adaptation of the scale.^12^, ^13^, ^14^ Whilst making cultural/language adaptation for ETDQ-7, initially two academicians carried out blind translations from English to Turkish. Thereafter, the accordance between two languages was checked by a linguist. Finally, the scale which was translated into Turkish was translated into English again by a bilingual academician. Ultimately, no meaning loss was detected among scale wording. Considering expert opinions, a final state for the scale was determined. Later on, as a pilot study, five patients were requested to fill out Turkish versions of the scale and asked if there were any cognitive dissonance to be corrected. Heeding their opinions, all obscure sentences in Turkish version of ETDQ-7 were enhanced to achieve comprehensibility. Then, the scale was applied to all participants. Two weeks after the first evaluation, 15 of the cases filled out the scale again without any treatment intervention.

### Statistical analysis

The data were evaluated with SPSS 20.0 package program for Windows (IBM Corporation, Armonk, New York, USA). Conformity of the data to normal distribution was estimated with the Kolmogorov–Smirnov test. Limiting value was specified as 20% in floor-ceiling effects.^15^ Paired sample *t* test from parametric tests was used to see if there was a difference in test–retest scores. Correlations between test–retest results were obtained with Pearson correlation analysis. Number, percentage, mean and Standard Deviation (SD) were listed as descriptive data. Independent sample *t* test was used to make comparison between item and total scores for the ETD and control groups in ETDQ-7 scale. Mann–Whitney *U* test was used to compare the ETD and control groups’ middle ear pressure. Known-groups method was used in validity analysis. Floor-ceiling effect, test–retest method, item-total score correlation and Cronbach's alpha coefficient as an internal consistency analysis were used in reliability analyses. For statistical significance level, *p*-value was accepted to be less than 0.05.

## Results

Twenty five (62.5%) patients of total 40 cases diagnosed with ETD were female and 15 (37.5%) were male. The mean age of the control group was 36.4 ± 10.2 (min 18, max 54) while 39.8 ± 11.8 years old (min 18, max 62) in the ETD group. In terms of age and gender, no statistically significant difference was detected between the ETD and control groups (*p* < 0.05). For both left and right ears, middle ear pressures in patients with ETD were found to be statistically significantly more negative compared to the control group (*p* < 0.001, Mann–Whitney *U* test). It was also detected that floor-ceiling effects of the 2nd and 3rd questions in the scale were above 20%. Cronbach's alpha coefficient was equal to 0.714 (very high) for the entire instrument. As seen from Table 1, none of the Cronbach alphas were greater than 0.714 if any item was deleted.

**Table 1:** Descriptive statistics, item-total score correlation coefficients and internal consistency results for ETD group.

Scale items	Mean score ± SD	Ceiling effect (%)	Floor effect (%)	Cronbach alpha if item deleted	Total score correlation^a^ (*r*)
Pressure in the ears (Item 1)	4.3 ± 1.7	5.0	17.5	0.688	0.567
Pain in the ears (Item 2)	3.1 ± 1.7	30.0	5.0	0.664	0.651
A feeling that your ears are clogged or “under water” (Item 3)	4.9 ± 1.8	7.5	27.5	0.695	0.547
Ear symptoms when you have a cold or sinusitis (Item 4)	4.2 ± 2.3	25.0	25.0	0.702	0.605
Crackling or popping sounds in the ears (Item 5)	4.1 ± 1.7	10.0	10.0	0.674	0.616
Ringing in the ear (Item 6)	3.5 ± 2.0	22.5	10.0	0.712	0.503
A feeling that your hearing is muffled (Item 7)	4.5 ± 1.9	10.0	22.5	0.617	0.784

a Pearson correlation test, all correlations are significant at *p* < 0.01 level.

The test–retest reliability coefficient for the total scale was determined as 0.792 (Table 2). There was no statistically significant difference detected while comparing first and last evaluation scores of the scale items in test–retest reliability except Item 2 (Table 3). In the ETD group, total and each item scores were found significantly higher than the control group (*p* < 0.001) (Table 4).

**Table 2:** Pearson correlation coefficient for each item in test–retest reliability.

Scale item no	*r*
Item 1 – retest Item 1	0.733^a^
Item 2 – retest Item 2	0.621^b^
Item 3 – retest Item 3	0.827^a^
Item 4 – retest Item 4	0.663^a^
Item 5 – retest Item 5	0.732^a^
Item 6 – retest Item 6	0.763^a^
Item 7 – retest Item 7	0.806^a^
Test total–retest total	0.792^a^

a Significant correlations at *p* < 0.01 level.

b Significant correlations at *p* < 0.05 level.

**Table 3:** Comparison of test–retest item score means.

Scale item no	Test score (*n* = 15) Mean ± SD	Retest score (*n* = 15) Mean ± SD	*t*	*p*^a^
Item 1	4.7 ± 1.3	4.4 ± 1.7	1.09	0.29
Item 2	3.6 ± 1.2	3.0 ± 0.9	2.35	0.03
Item 3	5.0 ± 1.9	4.7 ± 1.6	1.16	0.26
Item 4	5.0 ± 2.2	4.8 ± 1.8	0.59	0.56
Item 5	3.6 ± 1.7	3.8 ± 1.5	0.64	0.53
Item 6	3.2 ± 2.0	3.1 ± 1.7	0.38	0.70
Item 7	4.3 ± 1.9	4.2 ± 1.8	0.43	0.67
Total	29.7 ± 6.0	28.1 ± 5.2	1.65	0.12

a Paired sample *t* test.

**Table 4:** Comparison of ETD and control groups’ item scores.

Scale item no	ETD group score (*n* = 40) Mean ± SD	Control group score (*n* = 40) Mean ± SD	*t*	*p*^a^
Item 1	4.3 ± 1.7	1.1 ± 0.4	10.9	<0.001
Item 2	3.1 ± 1.7	1.2 ± 0.4	6.4	<0.001
Item 3	4.9 ± 1.8	1.2 ± 0.4	12.7	<0.001
Item 4	4.2 ± 2.3	1.3 ± 0.6	7.3	<0.001
Item 5	4.1 ± 1.7	1.1 ± 0.5	10.3	<0.001
Item 6	3.5 ± 2.0	1.2 ± 0.5	6.7	<0.001
Item 7	4.5 ± 1.9	1.1 ± 0.3	10.7	<0.001
Total	28.7 ± 8.2	8.5 ± 1.7	15.1	<0.001

a Independent sample *t* test.

## Discussion

There are substantial scales that are used routinely in the field of otorhinolaryngology. Sino-Nasal Outcome Test 22 (SNOT-22) is a frequently used Likert-type scale which evaluates complaints about nose and sinuses.^16^ Similarly, Otitis Media 6-Item Quality of Life Survey (OM-6) is used to test the quality of life for patients with chronic otitis media.^17^ McCoul et al. contributed ETDQ-7 as a disease specific Likert-type scale for Eustachian tube dysfunction in clinical practice. Authors used both OM-6 and SNOT-20 tests during the process of developing this scale.^4^

McCoul et al. stated that specificity and sensitivity of this scale for ETD was 100%. Although they prepared this scale with nine items initially, two of the questions were omitted owing to achieving a higher Cronbach's alpha with seven items and ETDQ-7 was finalized accordingly. Moreover, during internal consistency analysis Cronbach's alpha coefficient was found as 0.711.^4^ Consistent with these findings, Cronbach alpha coefficient was found as 0.714 in the present study. As Cronbach alpha coefficient approaches one, internal consistency reliability increases.^8^ The result achieved in the study asserts that the scale has internal consistency and has a high degree of reliability. Being a 7-item scale and its ease of application are ETDQ-7's most important advantages.^4^ Hence, it can be used in otorhinolaryngology practices and ETD-related researches prevalently. In 2014, its German validity and reliability study was done by Schroder et al.^18^ After implementing validity and reliability of the scale into different languages, the usage of the scale increased and was regarded as a quantitative method for investigating ETD in studies. Liu et al. stated that ETDQ-7 could be used as a helpful method for diagnosis in adults with serous otitis media.^6^ Van Roeyen et al. investigated value and discriminative power of ETDQ-7 and stated that ETDQ-7 is a beneficial disease-specific scale, whereas this test is not convenient for discriminating obstructive ETD and patulous Eustachian.^19^

Participants had difficulty in scoring Item 4 (“Increase in ear symptoms when you have a cold or sinusitis”) if they did not encounter these diseases over the last month during pilot study. Neither a note nor a criticism was detected in original scale or in validity and reliability edition of the scale. Thus, as authors, it is agreed to annotate a footnote under the related scale about this inquiry. For the Turkish version of the scale a footnote stating “if you do not complain about both diseases for the last month, please mark 1 (Not a problem)” was added.

The lowest mean score was detected for item 2 (3.1 ± 1.7) while Item 3 was marked as the highest (4.9 ± 1.8) in the scale. Furthermore, in Items 2 and 3, floor-ceiling effects were greater than 20%. Those items we suggest being interpreted carefully. These results showed similarities with analyses of McCoul et al. and Schroder et al. who investigated the German validity of the scale.^4^, ^18^ Thirty percentages of patients giving the lowest score (1/no problem) for Item 2 in the scale, “pain in ears” question, was a predictable outcome. It is possibly because many patients do not define earache in chronic ETD.^18^ In Item 3, where aural fullness was investigated, patients were asked if they had a feeling that their ears were clogged or “under water” and 27.5% of people noted it with highest score (7/severe problem) since this might be about being the most disturbing complaint for ETD. In the studies conducted by Park et al., ETD was denoted as the most common otologic disease among patients that applied to the hospital with aural fullness complaint.^20^

In our study, the test–retest reliability for overall test score was found as 0.792 (Table 2). Similarly, test–retest reliability was declared as 0.78 in McCoul et al.’s study.^4^ Both results indicate to us that reliability for the scale is relatively high for the tests applied at different times. Moreover, in our study, test–retest correlation value was detected to vary between 0.621 and 0.827. These results lead us to the fact that even if every item in the scale is answered by the same patient at different times, responses show consistency. Questions showed consistency over time both for total scale and for item.

Once each item and total score point averages obtained from control group (*n* = 40) were compared with ETD group, statistically significant difference was detected item and total score-wise (*p* < 0.001). Moreover, none of cases in control group had total scale scores greater than 14.5 which was stated as a threshold in McCoul et al. For all patients with ETD, total scale scores were observed to be greater than 14.5. These results might be interpreted such that while this scale is a valid discrimination between people with and without ETD, it also states that this scale is particular to ETD.

## Conclusion

In conclusion, a high-level of validity and reliability was determined in consequence of statistical analyses for Turkish version of Eustachian tube dysfunction questionnare-7. This scale is recommended for screening of ETD and evaluating severity of the disease.

## Conflicts of interest

The authors declare no conflicts of interest.

## References

[1] Shampo M.A., Kyle R.A. (1981). Bartolomeo Eustachi. JAMA.

[2] Bluestone C.D. (2005).

[3] Leuwer R., Koch U. (1999). Anatomy and physiology of the auditory tube. Therapeutic possibilities in chronic disorders of tubal function. HNO.

[4] McCoul E.D., Anand V.K., Christos P.J. (2012). Validating the clinical assessment of Eustachian tube dysfunction: the Eustachian Tube Dysfunction Questionnaire (ETDQ-7). Laryngoscope.

[5] Van Roeyen S., Van de Heyning P., Van Rompaey V. (2016). Responsiveness of the 7-item Eustachian Tube Dysfunction Questionnaire. J Int Adv Otol.

[6] Liu P., Su K., Zhu B., Wu Y., Shi H., Yin S. (2016). Detection of Eustachian tube openings by tubomanometry in adult otitis media with effusion. Eur Arch Otorhinolaryngol.

[7] McCoul E.D., Anand V.K. (2012). Eustachian tube balloon dilation surgery. Int Forum Allergy Rhinol.

[8] Mishel M., Brink P., Wood M.J. (1998). Advanced design in nursing research.

[9] Tavsancil E. (2002).

[10] Srivastava S., Gupta S., Singh A. (1993). Efficacy of various methods in evaluation of Eustachian tube function. Indian J Otolaryngol Head Neck Surg.

[11] Williams P.S. (1975). A tympanometric pressure swallow test for assessment of Eustachian tube function. Ann Otol Rhinol Laryngol.

[12] Guillemin F., Bombardier C., Beaton D. (1993). Cross-cultural adaptation of health-related quality of life measures: literature review and proposed guidelines. J Clin Epidemiol.

[13] Landgraf J.M., Maunsell E., Speechley K.N., Bullinger M., Campbell S., Abetz L. (1998). Canadian-French, German and UK versions of the Child Health Questionnaire: methodology and preliminary item scaling results. Qual Life Res.

[14] Ware J.E., Keller S.D., Gandek B., Brazier J.E., Sullivan M. (1995). Evaluating translations of health status questionnaires. Methods from the IQOLA project. International Quality of Life Assessment. Int J Technol Assess Health Care.

[15] GraphPad Software (2016). http://www.graphpad.com/guides/prism/6/statistics/index.htm?stat_skewness_and_kurtosis.htm.

[16] Rosenfeld R.M., Goldsmith A.J., Tetlus L., Balzano A. (1997). Quality of life for children with otitis media. Arch Otolaryngol Head Neck Surg.

[17] Schilder A.G., Bhutta M.F., Butler C.C., Holy C., Levine L.H., Kvaerner K.J. (2015). Eustachian tube dysfunction: consensus statement on definition, types, clinical presentation and diagnosis. Clin Otolaryngol.

[18] Schroder S., Lehmann M., Sudhoff H., Ebmeyer J. (2014). Assessment of chronic obstructive Eustachian tube dysfunction: evaluation of the German version of the Eustachian Tube Dysfunction Questionnaire. HNO.

[19] Van Roeyen S., Van de Heyning P., Van Rompaey V. (2015). Value and discriminative power of the seven-item Eustachian Tube Dysfunction Questionnaire. Laryngoscope.

[20] Park M.S., Lee H.Y., Kang H.M., Ryu E.W., Lee S.K., Yeo S.G. (2012). Clinical manifestations of aural fullness. Yonsei Med J.

